# Cost-effectiveness of therapeutic infant formulas for cow's milk protein allergy management

**DOI:** 10.3389/fnut.2023.1099462

**Published:** 2023-06-22

**Authors:** Narissara Suratannon, Panote Prapansilp, Athitaya Srinarongsook, Pornthep Tanpowpong, Pantipa Chatchatee, Krit Pongpirul

**Affiliations:** ^1^Center of Excellence for Allergy and Clinical Immunology, Division of Allergy, Immunology and Rheumatology, Department of Pediatrics, Faculty of Medicine, Chulalongkorn University, Bangkok, Thailand; ^2^King Chulalongkorn Memorial Hospital, The Thai Red Cross Society, Bangkok, Thailand; ^3^Medical Sciences, Reckitt | Mead Johnson Nutrition, Bangkok, Thailand; ^4^Department of Pediatrics, Faculty of Medicine, Ramathibodi Hospital, Bangkok, Thailand; ^5^Department of International Health, Johns Hopkins Bloomberg School of Public Health, Baltimore, MD, United States; ^6^Department of Preventive and Social Medicine, Faculty of Medicine, Chulalongkorn University, Bangkok, Thailand

**Keywords:** allergy, atopic disease, cow's milk protein allergy, atopic dermatitis, rhinitis, asthma, infant formula

## Abstract

Cow's milk protein allergy (CMPA) is children's most common food allergy. Therapeutic infant formulas for CMPA lead to symptom-free and potentially benefit early tolerance induction and reducing the allergic march in non-breastfed babies. This study assessed the cost-effectiveness of CMPA management with different therapeutic infant formulas in Thailand, which may reflect situations in developing countries throughout Asia. An analytic decision model was developed to simulate the occurrence of eczema, urticaria, asthma, rhinoconjunctivitis, or being symptom-free in infants with CMPA over 36 months. Extensively hydrolyzed casein formula with added probiotic *Lacticaseibacillus rhamnosus* (previously *Lactobacillus rhamnosus*) strain GG (EHCF+LGG), extensively hydrolyzed whey formula (EHWF), soy protein-based formula (SPF), and amino acid formula (AAF) were compared from the healthcare payer perspective. The results from a prospective cohort study were used for comparative effectiveness measures, while local experts were interviewed to estimate the healthcare resource used in the management of CMPA. The costs of healthcare resources were obtained from standard, publicly available sources. The direct medical cost of CMPA management was lowest for EHCF+LGG (USD 1,720), followed by SPF (USD 2,090), EHWF (USD 2,791), and AAF (USD 7,881). Compared with other formulas, EHCF+LGG was expected to save USD 370 (SPF), USD 1,071 (EHWF), and USD 6,161 (AAF) in the total cost of CMPA management over 36 months. In conclusion, EHCF+LGG was the most cost-effective strategy for managing non-breastfed infants with CMPA. This strategy was associated with more children developing immune tolerance to cow's milk and being symptom-free, contributing to overall cost-saving potential.

## Introduction

Cow's milk protein allergy (CMPA) is an immune-mediated hypersensitivity reaction to one or more cow's milk proteins. The typical allergic symptoms involve the skin (rash, eczema, and urticaria), the gastrointestinal tract (diarrhea, mucosal, and bloody stool), and the respiratory tract (wheezing and other breathing difficulties) (1). CMPA is a leading cause of food allergy in infants and children under 3 years of age, with an increasing prevalence in Thailand (2). CMPA was among the top three causes of food-induced anaphylaxis reported in a tertiary care hospital in Bangkok during 2008–2018 (3). Children with CMPA are at an increased risk of developing allergic march. They are five times more likely to have early-onset atopic dermatitis (4), which may lead to allergic rhinoconjunctivitis and asthma (5). They are also at an increased risk of poor growth, as a 2019 international survey demonstrates that cow's milk elimination leads to lower weight-for-height Z-scores than other food eliminations (6).

Based on the international consensus guidelines, the diagnosis of CMPA is based on medical and dietary history and confirmed through diagnostic elimination/oral food challenge. Specific IgE or skin prick tests on cow's milk might be performed when IgE-mediated reactions are suspected (1). The adequate management of CMPA resolves current allergic symptoms and prevents disease progression (5). The fundamental principle in treating CMPA is the dietary elimination of cow's milk protein (7).

As breastfeeding remains the primary recommended source of nutrition for infants with CMPA, cow's milk and dairy products should be restricted in the maternal diet because they may present in breast milk up to 7 days after consumption (8). However, in non-breastfed infants and children younger than 2 years, replacement with a therapeutic infant formula designed for CMPA is mandatory (7). For formula-fed infants, whey- or casein-based extensively hydrolyzed formula (eHF) that have short, cow's milk-derived peptides produced by multiple enzymatic processes has been demonstrated as the first-line management of CMPA (5, 7). Each eHF has peptides with different molecular weights and profiles (5). In children who have no co-allergy to soy, some doctors use the soy protein-based formula (SPF), as it is less expensive and has a better acceptance than eHF and amino acid formula (AAF). However, SPF is not hypoallergenic, contains phytate, aluminum, and phytoestrogens (isoflavones), is not recommended as the first choice for CMPA management (7, 9), and no current data demonstrate the use of SPF results in accelerated oral tolerance. AAF is recommended for a child with IgE-mediated CMPA at high risk of anaphylactic reactions when eHF cannot be used or multiple food intolerances. However, AAF does have numerous limitations, including high cost and the unlikelihood of oral tolerance development (7, 9).

In Thailand, EHCF with added probiotic *Lacticaseibacillus rhamnosus* (previously *Lactobacillus rhamnosus*) strain GG (EHCF+LGG) is the current standard for CMPA management practice among pediatricians and allergists. A prospective CMPA cohort study recently published in 2021 investigated the effect of different formulas on the occurrence of other allergic manifestations and the time of immune tolerance acquisition over 36 months. EHCF+LGG was associated with a lower incidence of allergic manifestations and a greater rate of immune tolerance acquisition. Infants with CMPA who received EHCF+LGG achieved early CMPA tolerance and had a higher chance of being symptom-free (10). Thus, EHCF+LGG formula can improve the long-term quality of life for infants with CMPA, as they can start consuming a regular diet, including dairy-containing products, earlier in life than other formulas. These improved clinical outcomes of infants with CMPA can potentially save the payer by decreasing direct medical costs due to faster tolerance acquisition and absence of symptoms, as well as parents' productivity loss due to frequent hospital visits (11).

Therapeutic infant formulas for CMPA management are not included in the country's universal health coverage scheme. The infant formulas are classified as over-the-counter products, and prices vary by the market mechanism. Hence, the parents must pay out of pocket, which limits the opportunities for the management of CMPA for many infants and children in Thailand. A previous cost-effectiveness analysis from the United Kingdom demonstrated that EHCF+LGG was the most cost-effective in reducing National Health Service resource use and improving CMPA tolerance compared with SPF, EHWF, and AAF. The current study aimed to assess the cost-effectiveness of CMPA management between therapeutic infant formulas in Thailand, which may reflect applicability in developing newly industrialized countries from the healthcare payer perspective. For reporting economic evaluations of health interventions, the Consolidated Health Economic Evaluation Reporting Standards (CHEERS) were used as a guide (12).

## Materials and methods

### Model

**Figure 1:**
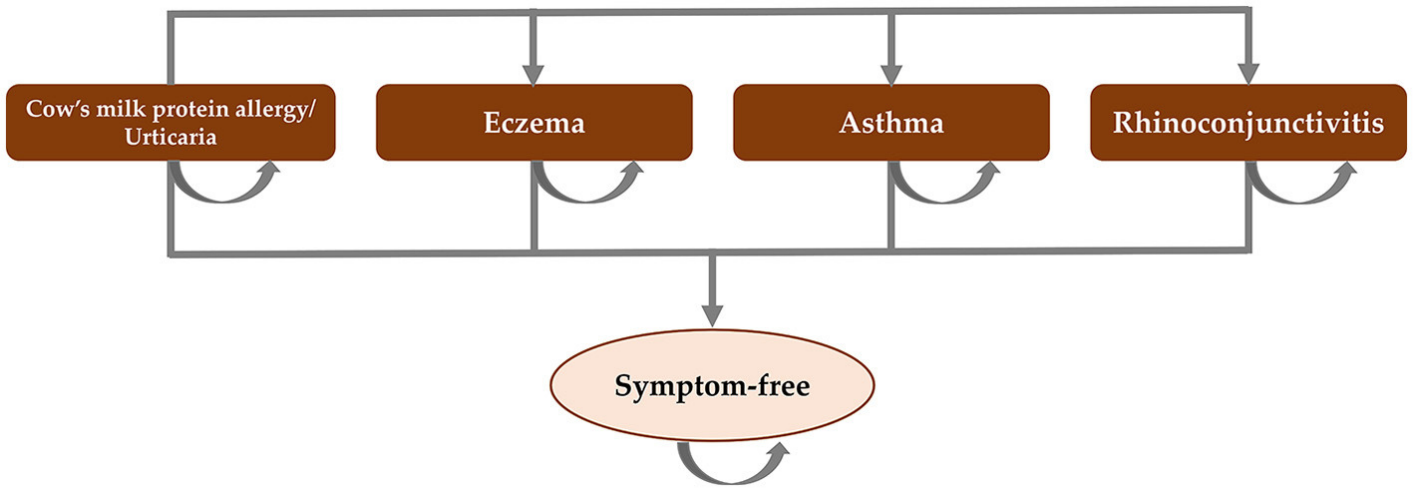
Model structure. Adapted from Martins et al. (11).

Cost-effectiveness analysis literature was reviewed across different regions. An annual decision analytic model was developed from the United Kingdom model (11), which was populated based on the atopic march cohort study (10), to simulate the occurrence of eczema, urticaria, asthma, rhinoconjunctivitis, or being symptom-free in infants with CMPA. Figure 1 presents the model structure.

The target population was 1–12-month-old infants with suspected IgE-mediated CMPA who needed therapeutic infant formulas. The cost-effectiveness of formula choices for CMPA management in Thailand, including EHCF+LGG extensively hydrolyzed whey formula (EHWF), SPF, and AAF, were compared from the Thailand healthcare payer perspective over 36 months to cover the period that most children with CMPA would likely need to use therapeutic infant formulas. Primary outcomes included total costs for the probability of symptom-free, probability of cow's milk tolerance, life years with cow milk tolerance, and life years with symptom-free. All children presented with urticaria and gastrointestinal symptoms for CMPA at the baseline and can either remain in this state or transition to have other allergic manifestations (rhinoconjunctivitis, asthma, or eczema).

The probability of developing symptoms and becoming tolerant to cow's milk protein was based on the previous comparative trial (10), and local key opinion leader interviews were used for comparative effectiveness measures in the model.

The frequency and amount of healthcare resources used to manage CMPA, and its allergic symptoms were informed by expert inputs from key opinion leaders of pediatricians and allergists practicing in the country. Total costs were calculated using Ministry of Public Health (MOPH) unit costs, MOPH median price of drugs, and published literature.

The analysis results are fully incremental, reported as cost per child tolerant to cow's milk protein or cost per symptom-free child.

### Assumptions

- Health states are exhaustive and mutually exclusive (i.e., one cannot occupy two states simultaneously, as we do not have data on children presenting with more than one allergic symptom at the same time).
- Children can develop cow's milk protein tolerance at any point of time.
- Once tolerant to cow's milk, one cannot go back to develop intolerance symptoms.

### Outcomes

The model inputs of the probability of allergic manifestations and immune tolerance acquisition were from a prospective cohort study conducted on 365 non-breastfed infants aged 1–12 months with suspected IgE-mediated CMPA who received EHCF+LGG, EHWF, SPF, AAF, or rice hydrolyzed formula (10).

For 36 months of follow-up, the use of EHCF+LGG for CMPA treatment is associated with a lower incidence of atopic manifestations (0.22, 0.09–0.34 in the EHCF+LGG cohort; 0.52, 0.37–0.67 in the rice hydrolyzed formula cohort; 0.58, 0.43–0.72 in the SPF cohort; 0.51, 0.36–0.66 in the EHWF cohort; and 0.77, 0.64–0.89 in the AAF cohort) and greater rate of immune tolerance acquisition than other formulas. Table 1 shows the probability results of the 36 months.

**Table 1:** Annual probabilities of allergic manifestations and immune tolerance acquisition for the 36-month atopic march cohort study were used for effectiveness measure.

**Formula**	**Time**	**Urticaria**	**Eczema**	**Asthma**	**Rhinoconjunctivitis**	**Symptom-free**	**CM Tolerance**
	Year 1	0.026	0.000	0.001	0.000	0.972	0.411
EHCF+LGG	Year 2	0.056	0.096	0.014	0.053	0.782	0.641
	Year 3	0.041	0.041	0.109	0.056	0.753	0.809
	Year 1	0.123	0.247	0.014	0.082	0.534	0.143
SPF	Year 2	0.097	0.054	0.082	0.095	0.671	0.226
	Year 3	0.027	0.069	0.192	0.152	0.559	0.399
	Year 1	0.081	0.220	0.083	0.082	0.535	0.195
EHWF	Year 2	0.055	0.014	0.055	0.069	0.807	0.314
	Year 3	0.083	0.055	0.138	0.152	0.572	0.425
	Year 1	0.151	0.289	0.00	0.178	0.381	0.016
AAF	Year 2	0.097	0.082	0.069	0.138	0.615	0.099
	Year 3	0.041	0.041	0.192	0.041	0.685	0.192

Annual probabilities for each allergic manifestation and immune tolerance were calculated using the difference in cumulative incidence in each year, and symptom-free probabilities were calculated using the 1-sum of all allergic manifestation incidence in each year over 36 months.

### Cost and resource use

The use of therapeutic infant formulas for CMPA in Thailand was estimated as 8, 10, 9, and 9 cans per month at 0–6, 6–12, 12–18, and 18–24 months of age, respectively. The amount of therapeutic formula does not differ with formula types by the expert opinion.

The estimated costs used to populate the model were from standard, publicly available sources. Direct medical costs associated with atopic diseases among young children were from the MOPH unit cost 2019 (13), the cost-of-illness study (14), and the standard cost list 2011 (15), as shown in Supplementary Table 1.

The estimated drug costs derived from the MOPH median price 2021 are presented in Supplementary Table 2 (16). Drug use posology was based on the electronic medicines compendium, an up-to-date, approved and regulated prescribing and patient information for licensed medicines websites (17). Supplementary Table 3 presents drug cost calculations.

The price of therapeutic infant formulas for the management of CMPA was based on the survey from the current clinical practice at the government and private hospitals, as shown in Supplementary Table 4. All costs and prices were converted from Thai Baht (THB) to United States Dollar (USD).

### Model results

The model was used to estimate total costs for the probability of symptom-free, probability of cow's milk tolerance, life years with cow milk tolerance, and life years with symptom-free.

The incremental cost-effectiveness ratios (ICERs) and net monetary benefit (NMB) were calculated, as the incremental cost divided by the incremental probabilities and life years of symptom-free and cow's milk tolerance at 3 years. NMB threshold in the current study was USD 600 and USD 4,800. USD 4,800 per Quality-Adjusted Life Year (QALY) is the Thai cost-effectiveness threshold. Discounted cost in 2 and 3 years was 3.5% per year as the literature reviewing following the guideline (11, 18). There was no willingness-to-pay (WTP) threshold for the CMPA treatment outcome in the model, so the minimum and maximum cost range per 3-year outcome was used.

### Sensitivity analyses

Deterministic sensitivity analysis was used to assess the uncertainty of the model. One-way sensitivity analysis was tested for the main drivers of the ICERs with life years, with symptom-free and cow's milk tolerance as the outcome. A Monte Carlo simulation accounts for parameter uncertainty by sampling 1,000 times from distributions assigned to model inputs. All parameters involved in the outcome, e.g., the probability of symptom-free or cow's milk tolerance of each allergic manifestation in each year, diagnostic procedure cost, service cost, and therapeutic infant formula price, were included as the drivers in the sensitivity analysis. The output variable was calculated for a new input variable under the unchanged assumptions.

## Results

### Base case

**Figure 2:**
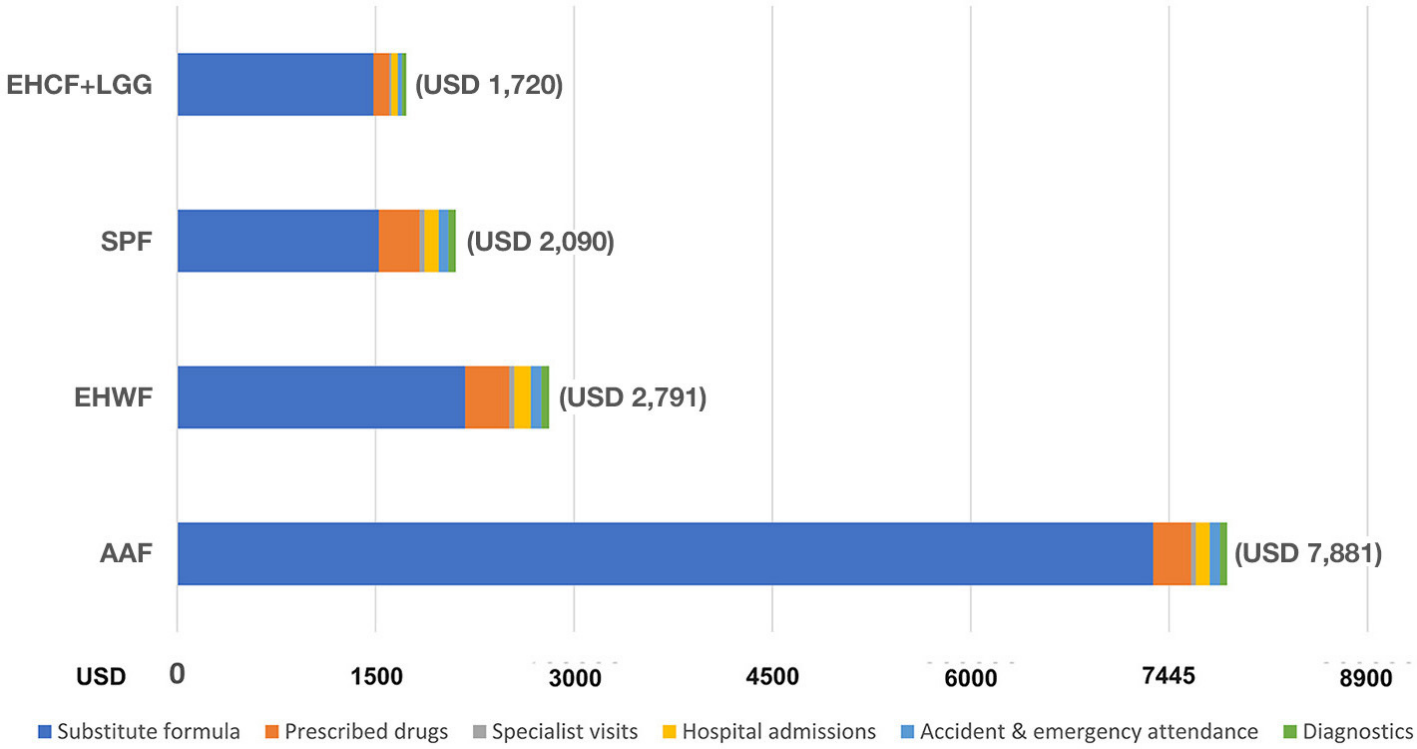
Total cost of CMPA management over 36 months.

As cost-effectiveness analysis in the base case, the infants who received EHCF+LGG had the lowest total medical cost of CMPA management over the 36 months (USD 1,720) compared with SPF (USD 2,090), EHWF (USD 2,791), and AAF (USD 7,881), as shown in Figure 2.

Therapeutic infant formula for CMPA constituted the most considerable portion. The total cost of clinical nutrition with EHCF+LGG was lower than with EHWF and AAF. The second highest cost was due to prescribed medication for atopic manifestations.

**Figure 3:**
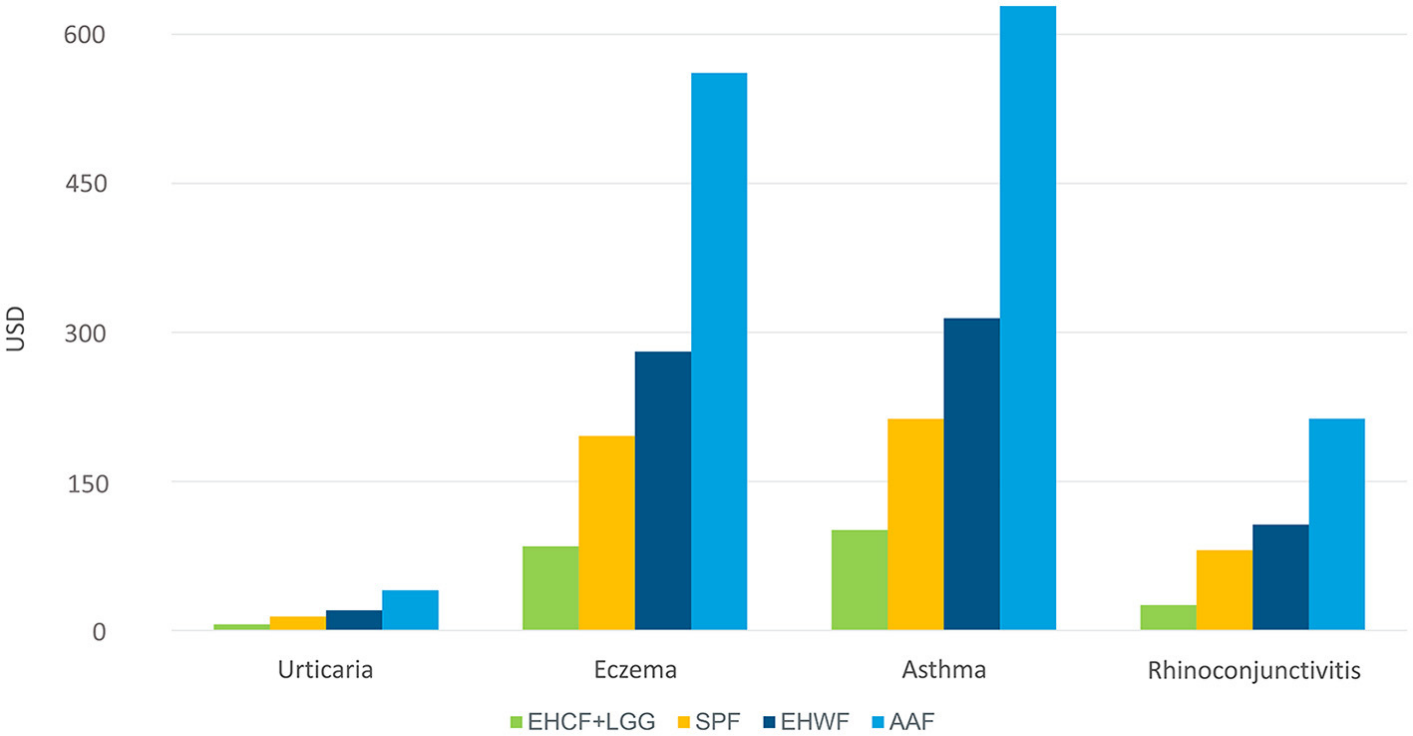
Cost of per management of atopic manifestations.

For the management of eczema and asthma, which were the top high cost per management of atopic manifestation, EHCF+LGG was associated with less healthcare resource use, as shown in Figure 3.

The infants who received EHCF+LGG also had the highest probability of symptom-free and cow's milk tolerance at a 36-month time horizon and the highest net monetary benefit with the lowest overall costs (dominant), compared with the infants who received SPF, EHWF, or AAF.

Extensively hydrolyzed casein formula with added probiotic *Lacticaseibacillus rhamnosus* strain GG (EHCF+LGG) still be dominant over all other comparators when cumulative life years with symptom-free and life years tolerant to cow's milk protein at 3 years were used as the denominators to the ICERs, as shown in Table 2.

**Table 2:** Deterministic results at 36 months for the probability of being symptom-free, probability of cow's milk tolerance, life years with symptom-free, and life years with cow's milk tolerance.

**Comparator**	**Total costs**	**Effects**	**Δ Costs**	**Δ Effect**	**ICER**	**Net monetary benefit**	
						**USD 600.00**	**USD 4,800.00**
**Probability of being symptom-free**							
EHCF+LGG	USD 1,701.41	0.753				–USD 1,252.94	USD 1,886.35
SPF	USD 2,033.74	0.586	USD 332.34	−0.167	Dominated	–USD 1,684.60	USD 759.44
EHWF	USD 2,733.75	0.559	USD 1,032.34	−0.194	Dominated	–USD 2,400.86	–USD 70.64
AAF	USD 7,830.36	0.572	USD 6,128.95	−0.181	Dominated	–USD 7,489.72	–USD 5,105.21
**Probability of cow's milk tolerance**							
EHCF+LGG	USD 1,701.41	0.755				–USD 1,251.67	USD 1,896.51
SPF	USD 2,033.74	0.384	USD 332.34	−0.371	Dominated	–USD 1,804.88	–USD 202.84
EHWF	USD 2,733.75	0.373	USD 1,032.34	−0.383	Dominated	–USD 2,511.89	–USD 958.85
AAF	USD 7,830.36	0.397	USD 6,128.95	−0.358	Dominated	–USD 7,594.07	–USD 5,940.03
**Life years with symptom-free**							
EHCF+LGG	USD 1,701.41	2.340				–USD 307.48	USD 9,450.02
SPF	USD 2,033.74	1.685	USD 332.34	−0.655	Dominated	–USD 1,030.13	USD 5,995.14
EHWF	USD 2,733.75	1.646	USD 1,032.34	−0.694	Dominated	–USD 1,753.15	USD 5,111.03
AAF	USD 7,830.36	1.786	USD 6,128.95	−0.554	Dominated	–USD 6,766.56	USD 680.01
**Life years with cow's milk tolerance**							
EHCF+LGG	USD 1,701.41	1.785				–USD 638.51	USD 6,801.73
SPF	USD 2,033.74	0.793	USD 332.34	−0.992	Dominated	–USD 1,561.66	USD 1,742.95
EHWF	USD 2,733.75	0.734	USD 1,032.34	−1.051	Dominated	–USD 2,296.88	USD 761.20
AAF	USD 7,830.36	0.895	USD 6,128.95	−0.889	Dominated	–USD 7,297.18	–USD 3,564.93

An ICER is calculated by dividing the incremental cost by the incremental health effect, yielding a cost-per-unit-of-health-effect ratio. Dominated is defined as an intervention with higher costs and worse outcomes than an alternative intervention. The net monetary benefit is determined by converting the health outcome into monetary terms based a willingness to pay threshold.

**Figure 4:**
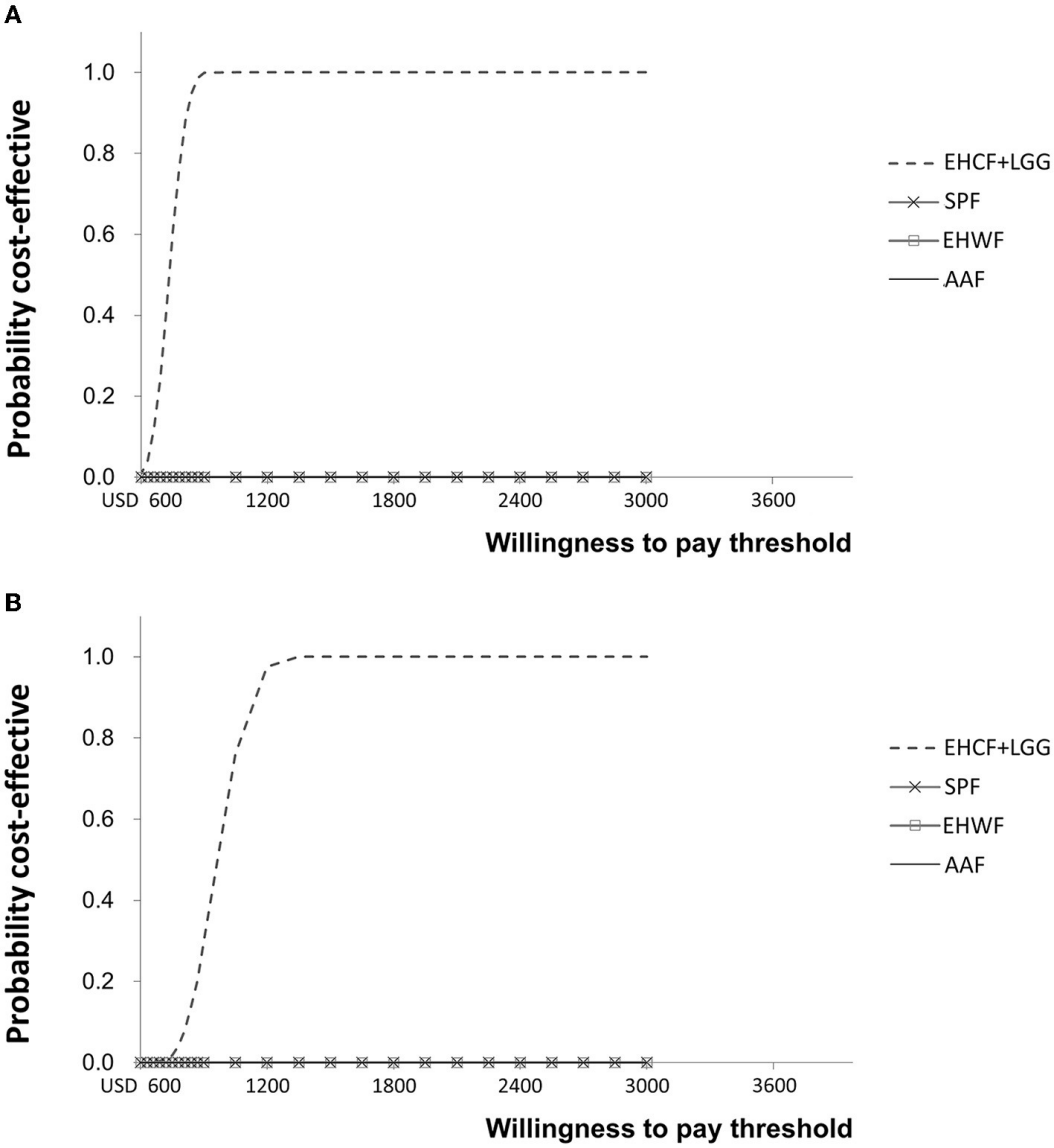
Cost-effectiveness acceptability curve for life years with symptom-free **(A)** and life years with cow's milk tolerance **(B)**.

According to probabilistic analysis, the cost-effectiveness acceptability curve for life years with symptom-free and life years with cow's milk tolerance showed that EHCF+LGG had the highest probability of being cost-effective compared with other formulas, as shown in Figure 4. EHCF+LGG also had the highest net monetary benefit at a WTP of USD 600 and USD 4,800 per additional child living symptom-free or tolerant to cow's milk.

### Sensitivity analysis

A one-way sensitivity analysis was used, with the tornado diagram reflecting the variation around the ICER from the 10 most influential parameters changing to the lower and upper bounds of their 95% confidence intervals. The extensively hydrolyzed formula, the first-line management option for mild-to-moderate CMPA, EHCF+LGG, and EHWF, was compared. ICERs were negative values because EHCF+LGG dominates the comparators.

**Figure 5:**
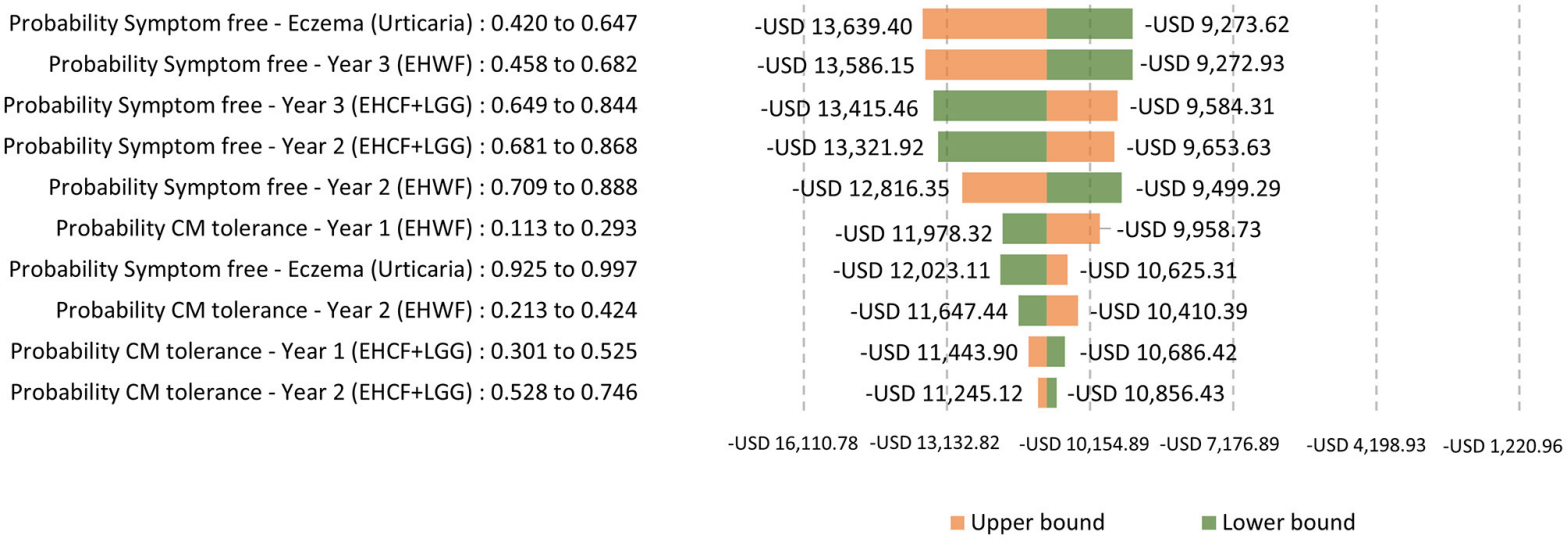
One-way sensitivity analysis: Testing for the main drivers of the ICER with life years with symptom-free as the outcome.

**Figure 6:**
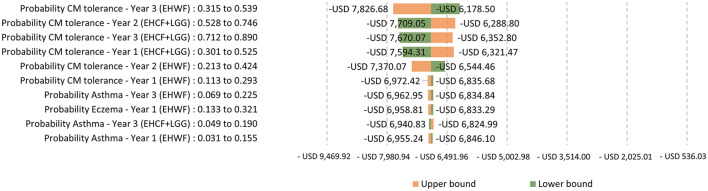
One-way sensitivity analysis: Testing for the main drivers of the ICER with life years with cow's milk tolerance as the outcome.

Testing for the main drivers of the ICER with life years with symptom-free as the outcome, the ICERs were sensitive to the change in the probability of being symptom-free and cow's milk tolerant. The top parameter is the most likely to affect the model's ICER outcome or predictions. The probability of symptom-free eczema was the most sensitive, as shown in Figure 5. Testing for the main drivers of the ICER with life years with cow's milk tolerance as the outcome, the probability of being tolerant was sensitive to the change, as shown in Figure 6.

## Discussion

Similar to the previously reported cost-effectiveness analyses (11, 18), those reported here demonstrated ECHF+LGG as the most cost-effective strategy to manage non-breastfed infants with CMPA in Thailand. A recent study from Indonesia, using the private payers' perspective, also demonstrated that EHCF+LGG was the most cost-effective strategy when compared to EHWF and AAF and to be more effective and saved healthcare resources when compared with SPF (19). EHCF+LGG may provide a higher chance of developing immune tolerance to cow's milk and being symptom-free, thus contributing to the high-cost saving potential. EHCF+LGG is associated with the lowest cost of eczema and asthma management. Furthermore, the short duration of CMPA management by effective therapeutic infant formulas such as EHCF+LGG can help save the consequential expense and especially hospital bed quota for patients experiencing severe symptoms, thus benefiting the public health system through budget saving.

The results of this study show that therapeutic infant formulas and prescribed drugs are the top two high-cost clinical resources for CMPA treatment and the associated conditions, respectively. These findings are consistent with a health economic study on CMPA infants with proctocolitis and eczema in Turkey, which found that clinical nutrition was the primary cost driver, accounting for 89–92% of total direct medical costs over a 2-year period. Management of CMPA infants with eczema and/or exclusively formula-fed infants was associated with higher total direct medical costs from both payer and societal perspectives (20). Unfortunately, there is no universal coverage of eHF and some eczema medications; thus, the caregivers must bear their expenses.

While formula price is not among the top influential factors in determining the most cost-effective strategy for managing CMPA in sensitivity analysis, it is still perceived as crucial by price-sensitive consumers. Formula cost represents an up-front cost that is more immediate, and thus apparent, than the long-term saving potential, along with the fact that therapeutic infant formulas are high cost compared with their non-therapeutic infant counterparts, the general consumer may choose the lower cost alternatives. This current study may improve the consumer perception of the EHCF+LGG formula as a long-term, cost-effective strategy.

The estimated costs used in this study may be lower than depicted. For example, surveyed prices of the therapeutic infant formulas for CMPA may be lower than the actual prices that consumers have to pay, which change over time due to many factors, including inflation, manufacturing, and demand-supply in the market. Similarly, specialist visit costs and drug costs in the present research may be lower than actual costs, as these numbers were sourced from the average MOPH-linked hospitals that mostly used generic drugs and the strategy used for the therapeutic infant formula(s) for managing cases with CMPA. All direct medical costs associated with atopic diseases were adjusted for inflation to reflect prices in 2021. The net monetary benefit threshold of USD 4,800 (as shown and discussed in Table 2) is also the summary statistic reflecting the largest amount of money MOPH is willing to spend on an intervention to improve 3 years of life. The cost-effectiveness analysis of therapeutic infant formulas for managing CMPA in the United Kingdom and Indonesia revealed that, like Thailand, EHCF+LGG was likely the most cost-effective formula option regarding the cost-effectiveness strategy (11, 19). As the results of one-way sensitivity analysis, the cost-effectiveness of the strategy for the ICER with life years with symptom-free or life years with cow's milk tolerance may be changed if the probability of symptom-free and CM tolerance varied.

There are some limitations in this study. First, the model is based on data from a non-randomized cohort study conducted in a single European country, as there is no cohort study in the Asia-Pacific region. The current study did not include the rice hydrolyzed formula group because it either has not been recommended for CMPA management or is available in the country. Thus, we used the input parameters such as formula price, direct medical cost, drug cost, and resources for CMPA management that are locally derived to reflect and benefit countries with similar variables. Second, allergic manifestation diagnosis criteria may differ compared between studies.

## Conclusion

EHCF+LGG formula was the most cost-effective strategy for non-breastfed infants and children with CMPA. EHCF+LGG is associated with more children developing immune tolerance to cow's milk and being symptom-free, yielding the most significant cost-saving potential *via* lower long-term medical costs and subsequent decrease of the associated economic burden of disease on the healthcare system.

## Data availability statement

The original contributions presented in the study are included in the article/Supplementary material, further inquiries can be directed to the corresponding author.

## Author contributions

PP, PC, and KP: conceptualization. KP: methodology and project administration. AS and KP: software, formal analysis, and writing—original draft preparation. AS, NS, PT, and PC: validation. AS: investigation. PP: resources and funding acquisition. AS, NS, and PC: data curation. PP, AS, and KP: writing—reviewing and editing. NS and PT: visualization. PP and PC: supervision. All authors have read and agreed to the published version of the manuscript.
