# Supplementary material for: Cost-effectiveness of therapeutic infant formulas for cow's milk protein allergy management
Source: Front Nutr. 2023 Jun 22;10:1099462. doi: 10.3389/fnut.2023.1099462 (PMC10281430; doi:10.3389/fnut.2023.1099462)
Supplement: Supplementary file 1 [file Data_Sheet_1.docx]

Supplementary tables

**Supplementary Table 1.** Direct medical costs associated with atopic diseases among young children in Thailand.

|  | **Cost* (USD)** | **Remarks** |
| --- | --- | --- |
| Initial specialist visit^a^ | 8.80 | Cost per visit |
| Follow-up specialist visit^a^ | 8.80 | Cost per visit |
| Accident & emergency attendance^b^ | 149.93 | Weighted average cost per AD episode |
| Hospital admission^b^ | 199.75 | Weighted average cost per AD episode |
| IgE blood test^a^ | 11.16 | Cost per test |
| Skin prick test^c^ | 15.95 | Cost per test |

* All costs were adjusted for inflation to reflect the costs in 2021.

Costs were sourced from ^a^ Thai Ministry of Public Health unit cost 2019; ^b^ the cost-of-illness study 2012; ^c^ Thai standard cost list 2011

**Supplement Table 2.** Drug costs for treatment of allergic manifestations involved short- and long-term use of topical and systemic medications based Thai Ministry of Public Health median price 2021.

| **Prescription^a^** | **Cost^b^ per month (USD)** |
| --- | --- |
| Systemic corticosteroids | 0.10 |
| Topical corticosteroids | 0.73 |
| Calamine lotion | 0.45 |
| Topical calcineurin inhibitor | 29.04 |
| Antihistamines | 0.29 |
| Montelukast sodium oral powder | 17.21 |
| Inhaled corticosteroids | 6.01 |
| Inhaled bronchodilator | 1.36 |
| Intranasal steroid spray | 13.86 |

^a^ Prescribed medications based on expert inputs.

**^b^** Drug costs were sourced from Thai Ministry of Public Health median price (announced 19 May 2021)

**Supplemental Table 3.** Drug cost calculations.

| **Drug** | **Cost for model** | **Units per package** | **Dose per unit** | **Cost per package^a^** | **Posology electronic medicines**  **compendium** | **Estimated amount used** |
| --- | --- | --- | --- | --- | --- | --- |
| **Systemic corticosteroids** | **USD 0.10** |  |  |  |  |  |
| Prednisolone tab 5 mg (10 tabs) | USD 0.10 | 500 tabs | 5 mg | USD 5.24 | Pediatric dose: 1 mg/kg/d for 5 days. Weight range from birth to 2 years (z-score) weight range: 5 to 17 kg | 5 to 15 mg for 3 days |
| **Topical corticosteroids*** | **USD 0.73** |  |  |  |  |  |
| Betamethasone valerate cream 0.1% (15 g) | USD 0.48 | 15 g | 0.10% | USD 0.48 | Short term use for symptom relief | 15 g for 1 week |
| Clobetasol propionate cream 0.05% (5 g) | USD 0.99 | 5 g | 0.05% | USD 0.33 |  |  |
| **Calamine lotion** | **USD 0.45** |  |  |  |  |  |
| Calamine lotion | USD 0.45 | 60 ml |  | USD 0.45 | Short term use for symptom relief | 1 bottle per month |
| **Calcineurin inhibitor** | **USD 29.04** |  |  |  |  |  |
| Tacrolimus oint 0.03% | USD 29.04 | 10 g | 0.03% | USD 29.04 | Apply thinly to twice daily for up to 3 weeks | 1 tube per month |
| **Antihistamines*** | **USD 0.29** |  |  |  |  |  |
| Cetirizine hydrochloride 5mg/5ml | USD 0.36 | 60 ml | 5 mg | USD 0.36 | 2.5 ml daily | 1 bottle for 3 to 5 days |
| Chlorpheniramine maleate syr 2 mg/5ml | USD 0.22 | 60 ml | 2 mg | USD 0.22 | 2.5 ml twice daily |  |
| **Montelukast sodium oral powder** | **USD 17.21** |  |  |  |  |  |
| Montelukast sodium oral powder 4 mg (30s) | USD 17.21 | 1 sachet | 4 mg | USD 0.57 | 1 sachet daily |  |
| **Inhaled corticosteroids*** | **USD 6.01** |  |  |  |  |  |
| Fluticasone propionate MDI 125 mcg/dose | USD 6.66 | 120 doses | 125 mcg | USD 6.66 | 100–250 micrograms twice daily, dose to be adjusted as necessary. | Assumed 2 puffs daily |
| Budesonide MDI 100 mcg/dose | USD 5.36 | 200 doses | 100 mcg | USD 5.36 |  |  |
| **Salbutamol** | **USD 1.36** |  |  |  |  |  |
| Salbutamol sulfate MDI 100 mcg/1 dose | USD 1.36 | 200 doses | 100 mcg | USD 1.36 | 100–20 micrograms, up to 4 times a day for persistent symptoms. | Assumed 200 mcg 4 times daily |
| **Intranasal steroid** | **USD 13.86** |  |  |  |  |  |
| Mometasone furoate nasal spray 50 mcg/dose | USD 13.86 | 60 doses | 50 mcg | USD 13.86 | 1 spray to each nostril once daily. | Mometasone furoate nasal spray 50 mcg/dose |

* Treatments assumed to be equally distributed due to absence of data.

^a^ Drug costs were sourced from Thai Ministry of Public Health median price (announced 19 May 2021)

**Supplement Table 4.** Costs of the therapeutic infant formulas for CMPA management based on current Thai clinical practice.

| **Formula type** | **Retail Selling Price* to the hospital 2022**  **Average (USD)** |
| --- | --- |
| EHCF + LGG | 16.97 |
| SPF | 11.08 |
| EHWF | 15.49 |
| AAF | 56.85 |

* Per 400 mg. Price were sourced from suggested retailed price of government and private hospital Retail Selling Price survey in 2022

Abbreviations: EHCF+LGG, extensively hydrolyzed casein formula with added probiotic Lacticaseibacillus rhamnosus strain GG; EHWF, extensively hydrolyzed whey formula, SPF, soy protein-based formula; AAF, amino acid formula
